# A Case of Purple Urine Bag Syndrome (PUBS) in a Patient With a Chronic Indwelling Foley Catheter

**DOI:** 10.7759/cureus.67731

**Published:** 2024-08-25

**Authors:** Kelsey Murray, Kishan Patel, James Espinosa, Alan Lucerna

**Affiliations:** 1 Emergency Medicine, Jefferson Health, Stratford, USA

**Keywords:** bacteria associated with purple bag syndrome, pathophysiology of purple bag syndrome, risk factors for purple bag syndrome, purple urine bag syndrome diagnosed in the emergency department, purple urine bag syndrome

## Abstract

Purple urine bag syndrome (PUBS) is a medical condition characterized by the appearance of purple discoloration in the urine collection bag of patients who use catheters for urinary drainage. PUBS is primarily seen in elderly, female, and institutionalized patients who have chronic indwelling catheters. The discoloration occurs due to the presence of certain bacteria that can produce indigo (which has a blue hue) and indirubin (with a red hue) leading to the formation of a purple color. PUBS may also indicate an underlying urinary tract infection or other medical conditions that require immediate attention. This case report provides an overview of PUBS, including its etiology, pathogenesis, clinical manifestations, and management. Early identification and appropriate management of PUBS can significantly improve patient outcomes, decrease healthcare costs, and enhance the overall quality of care provided to patients.

## Introduction

The typical yellow color of urine is due to urochrome as well as to urobilin and uroerythrin. Changes from this color can be distressing for patients, family members as well as care providers [1-4]. A purple discoloration of urinary drainage bags in the context of a urinary tract infection was first described in 1978 and has been termed Purple Urine Bag Syndrome (PUBS) [5]. In the years since the first description, PUBS has been known to be more commonly seen in elderly patients with chronic urethral catheterization and a urinary tract infection (UTI) or colonization, often in the setting of chronic constipation [5-6]. The syndrome is said to predominantly affect female patients [7]. The presence of alkaline urine has been proposed as a risk factor. However, the syndrome clearly can occur with acidic urine [4-7]. The overall incidence of PUBS in patients with long-term indwelling catheters varies widely in the literature but may be in the range of 8 to 9% [8-9]. PUBS has been reported in a patient with a nephrostomy tube and collection bag [10].

This case report was presented in poster form at the Rowan University Research Day, Stratford NJ, on May 7, 2023.

## Case presentation

**Figure 1:**
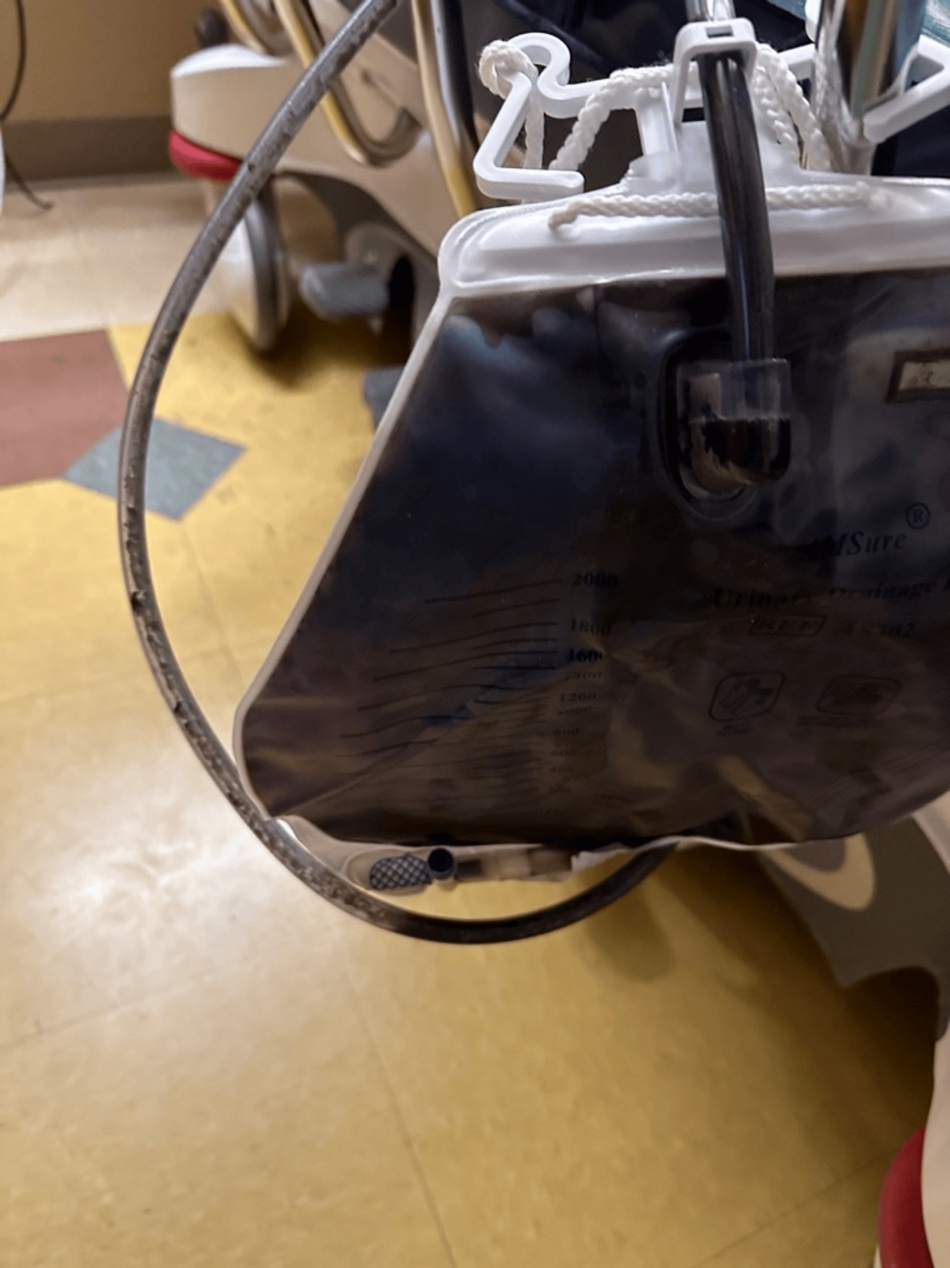
Foley bag showing purple color urine

A 46-year-old female with a history of cerebral palsy with a chronic indwelling Foley catheter, chronic UTIs, breast cancer with mastectomy, deep vein thrombosis (DVT), and osteomyelitis presented to the emergency department (ED) with complaints of a blocked Foley catheter. The patient reported noticing leakage around the catheter four days prior to ED presentation and observed a purple discoloration in the catheter bag two days prior to ED presentation. The patient had a prior history of E. coli species on urinary cultures and had been scheduled for a catheter replacement. Upon arrival, the patient's vital signs were stable, with a blood pressure of 159/83, heart rate of 79 beats per minute, temperature of 97.9°F, respiratory rate of 18, and SPO2 of 98% on room air. Physical examination revealed a non-toxic, well-appearing female with an indwelling Foley catheter. The tubing was discolored with white sediment crystals, likely the cause of the obstruction, as well as a purple film lining the tubing. The entire Foley bag was filled with purple-colored urine (Figure 1). The patient was discharged home on a prescription for sulfamethoxazole/trimethoprim.

The patient's lab results are listed in Table 1. The urine culture showed E. coli sp. sensitive to cephalexin and resistant to sulfamethoxazole/trimethoprim. The patient was notified at 48 hours after ED discharge and the patient's prescription was changed to cephalexin. At that time, the patient reported no recurrence of the purple-colored urine.

**Table 1:** Laboratory results

Laboratory Results	Result	Normal range	Units
White blood cell count	11.2	4.0-11.0	K/uL
Hemoglobin	11.3	10.6-15.6	g/dL
Platelet count	180.0	150-400	K/uL
Sodium	137.0	135-154	mEq/L
Potassium	3.6	3.5-5	mEq/L
BUN	18.0	5 to 20	mg/dL
Creatinine	1.0	0.6-1.2	mg/dL
Glucose	95.0	70-100	mg/dL
Calcium	10.2	8.5-10.5	mg/dL
Chloride	101	95-105	mEq/L
Bicarbonate	27	23-29	mEq/L
Urine color	brown	yellow	NA
Urine clarity	cloudy	clear	NA
Urine specific gravity	1.012	1.005-1.030	NA
Urine pH	7	5 to 7.5	NA
Urine glucose	negative	negative	NA
Urine protein	2+	negative	NA
Urine bilirubin	negative	negative	NA
Urine urobilinogen	negative	negative	NA
Urine ketones	1+	negative	NA
Urine blood	3+	negative	NA
Urine white cells	>100	0-5/HPF	cells/HPF
Urine red cells	>100	0-5/HPF	cells/HPF
Urine nitrite	negative	negative	NA
Urine leukocyte esterase	3+	negative	NA
Urine culture	>100,000 E. coli species and mixed flora	no growth or <10K CFU	NA

## Discussion

The purple hue of PUBS is due to a chemical cascade, in which intestinal bacteria metabolize tryptophan to indole. Indole is conjugated by the liver to indoxyl sulfate. Indoxyl sulfate is then excreted in the urine. However, some bacteria produce enzymes (indoxyl sulfatase and phosphatase) which can generate indigo (which has a blue hue) and indirubin (with a red hue). These two colors can combine to produce a purple precipitate on a urinary catheter and a urinary catheter bag [11]. Some gram-negative bacteria produce indoxyl sulfatase and phosphatase, including *E. coli, K. pneumonia, Proteus mirabilis, M. morganii, *and* Providencia stuartii *and* rettgeri*. *Enterococci *and* Group B Streptococci* have also been reported [11-12]. Bacterial subtypes within the same species of such common urinary tract pathogens as E. coli may not produce sulfatase or phosphatase. This may explain the rarity of PUBS despite the overall frequency of UTIs [13-14]. The time frame from placement of the urinary catheter to the development of PUBS has been reported as hours to a few days [7].

The pathophysiology of the cascade from tryptophan metabolism to the eventual urinary color change sheds light on the associated risk factors. Chronic constipation can both alter gut motility as well as favor the development of intestinal bacteria that are responsible for the first step of the process, which is the metabolization of tryptophan to indole through tryptophanase [7]. A diet that is high in tryptophan-containing foods may predispose to PUBS [13]. An alkaline urine environment is the general downstream result of this metabolic process [7]. The relatively higher incidence in female patients has been hypothesized to be due to shorter urethral length in females which may predispose to a UTI [7]. Debilitated patients with chronic indwelling Foley catheters appear to be more likely to develop PUBS than younger, more active patients with a chronic catheter. This has been hypothesized to be due to the relationship to an increased likelihood of constipation in a less active patient [7]. Renal failure has been described as a risk factor and has been hypothesized to be due to impaired clearance of indoxyl sulfate [2]. Dehydration can increase the concentration of any urinary indigo or indirubin that has been generated, making a purple color change more likely [3]. The plastic composition of urinary catheters and bags may predispose to a deeper color change [4]. However, the specific type and brand of catheter and bag may not make a difference in the process of color change [7-8].

The diagnosis of PUBS is essentially visual. Kalsi et al. point out that there have been no other causes reported for purple urine other than PUBS [3]. The color change generally indicates an underlying UTI or colonization [15]. Urine culture results will guide specific antibiotic therapy. Based on the clinical context, antibiotics may not be indicated. The urinary catheter and bag should be changed. Management of any related constipation is important [2].

Patients who have had PUBS may need to have their urethral catheters changed on a more frequent basis [7,9,11]. Hygiene optimization is a preventive element [15]. Prevention includes management of constipation [7,9]. Wang et al. describe a patient with PUBS with an associated UTI in which bladder function training was conducting in the daytime (with intermittent clamping) in order to simulate normal bladder filling and drainage. The patient showed improved bladder function, leading to the conversion to clean intermittent catheterization which has a lower risk of a UTI than an indwelling catheter [16].

PUBS has been discussed as an essentially benign process [2,5]. However, PUBS has been associated with poor outcomes in a number of case reports. Catheters can be associated with benign colonization requiring only changing the catheter. Severe catheter-related infections require a catheter change as well as antibiotic treatment. The difference is the clinical context. Tasi et al. presented two cases of PUBS syndrome with pyuria in which the patients progressed to Fourier's gangrene [17]. Bhattarai et al. presented a case of PUBS in a patient with a nephrostomy tube who progressed to sepsis [18]. In these cases, PUBS was seen. However, the outcome appears to have been related to the risk of indwelling cauterization and not to the PUBS process.

In the patient presented, the ability to make an early diagnosis of PUBS allowed for the patient to be discharged from the ED.

## Conclusions

Purple urine bag syndrome (PUBS) is a rare phenomenon which can cause significant anxiety for both patients and treatment teams, leading to unnecessary workup and treatment. By being aware of this condition, healthcare professionals can effectively manage patients with PUBS and provide reassurance to patients and their families.
